# Do Lifestyle Interventions in Pregnant Women with Overweight or Obesity Have an Effect on Neonatal Adiposity? A Systematic Review with Meta-Analysis

**DOI:** 10.3390/nu13061903

**Published:** 2021-06-01

**Authors:** Naiara F. Baroni, Nayara R. Baldoni, Geisa C. S. Alves, Lívia C. Crivellenti, Giordana C. Braga, Daniela S. Sartorelli

**Affiliations:** 1 Graduate Program in Public Health, Ribeirão Preto Medical School, University of São Paulo, Ribeirão Preto 14049-900, SP, Brazil; naiarabaroni@gmail.com (N.F.B.); iacrivelenti@gmail.com (L.C.C.); giordanacb@gmail.com (G.C.B.); 2 Graduate Program in Health Sciences, Federal University of São João del-Rei, São João del-Rei 36307-352, MG, Brazil; nrbaldoni@gmail.com (N.R.B.); geisa.cristina@gmail.com (G.C.S.A.); 3 Undergraduate Course in Nutrition, Itaúna University, Itaúna 35680-142, MG, Brazil; 4 Undergraduate Course in Nursing, Itaúna University, Itaúna 35680-142, MG, Brazil; 5 Department of Social Medicine, Ribeirão Preto Medical School, University of São Paulo, Ribeirão Preto 14049-900, SP, Brazil

**Keywords:** pregnant women, intervention, lifestyle, newborn, adiposity, childhood obesity

## Abstract

Excessive body fat at birth is a risk factor for the development of childhood obesity. The aim of the present systematic review with meta-analysis was to evaluate the effect of lifestyle interventions in pregnant women with overweight or obesity on neonatal adiposity. The PubMed, Embase, Web of Science, Scopus, and LILACS databases were used as information sources. Original articles from randomized clinical trials of lifestyle intervention studies on pregnant women with excessive body weight and the effect on neonatal adiposity were considered eligible. The risk of bias was assessed using Cochrane criteria. The meta-analysis was calculated using the inverse variance for continuous data expressed as mean difference (MD), using the random effect model with a 95% confidence interval (CI). The outcomes were submitted to the GRADE evaluation. Of 2877 studies, four were included in the qualitative and quantitative synthesis (*n* = 1494). All studies were conducted in developed countries, with three including pregnant women with overweight or obesity, and one only pregnant women with obesity. The interventions had no effect on neonatal adiposity [Heterogeneity = 56%, MD = −0.21, CI = (−0.92, 0.50)] with low confidence in the evidence, according to GRADE. Studies are needed in low- and medium-developed countries with different ethnic-racial populations. PROSPERO (CRD42020152489).

## 1. Introduction

The prevalence of childhood obesity is characterized as a pandemic and constitutes one of the greatest public health challenges of the 21st century [1]. In the period from 2000 to 2018, the number of children with overweight or obesity up to five years of age worldwide increased from 30.1 to 40.1 million, an increase of 33.2% in the period [2,3]. According to the World Health Organization (WHO), approximately 340 million children and adolescents aged 5–19 years were overweight in 2016 [4].

Increasing trends in child overweight are taking place in most world regions, not only in high-income countries, where prevalence is the highest (15% in 2011). The epidemic has been growing most rapidly in low- and middle-income countries, particularly in Northern and Southern Africa, the Middle East, and the Pacific Islands. In Africa, the estimated prevalence of overweight in children younger than 5 years increased from 4% in 1990 to 7% in 2011 and is expected to reach 11% in 2025. The prevalence of overweight is lower in Asia (5% in 2011), but the number of affected children is higher compared with Africa (17 and 12 million, respectively). A systematic review showed that in Latin America, the prevalence of overweight in children aged 0–19 years is about 25% [5,6,7].

Excess body weight can have immediate consequences for the physical and mental health of children and adolescents. It is considered a risk factor for developing chronic non-communicable diseases, such as obesity, diabetes, and cardiovascular diseases, culminating in premature death in adulthood. In addition, childhood obesity can negatively influence children’s learning and socialization in the school environment due to the stigma associated with being overweight [8,9].

Body composition at birth is an intrauterine growth marker [10]. The excessive accumulation of body fat at birth is considered a risk factor for the development of childhood obesity [11]. Data from observational studies suggest a positive association between the maternal preconception Body Mass Index (BMI) and the child’s birth weight and body composition parameters [12,13]. On the other hand, adequate weight gain during pregnancy promotes a better balance in the production of maternal adiponectin and leptin, important adipokines involved in the neonate’s metabolic programming for adiposity [13,14].

Opportunities to prevent and control obesity, and the chronic diseases associated with being overweight, occur during multiple stages of life. However, early interventions provide better chances of prevention, including prenatal and postnatal care, maternal nutrition, and the reduction of environmental exposure to risk factors even in the intrauterine period, a critical stage of development for health throughout life [15,16].

Evidence suggests that preconception maternal excessive body weight is associated with greater adiposity in children [17]. Recent studies have investigated the developmental origins of health and disease [18,19,20,21,22,23,24,25], with the adiposity of the newborn being a significant predictor of morbidity in the neonatal period and throughout life [26]. This review aimed to assess the effect of lifestyle interventions in pregnant women with overweight or obesity on neonatal adiposity. Additionally, birth weight, neonatal fat-free mass, and gestational weight gain were evaluated as secondary outcomes.

## 2. Materials and Methods

The Preferred Reporting Items for Systematic Reviews and Meta-Analyses (PRISMA) guidelines (Supplementary Tables S1 and S2) [27] and Cochrane Handbook for Systematic Reviews of Interventions, v6.1, UK [28], were used to develop the present study. The study was registered on the International Prospective Registry of Systematic Reviews—PROSPERO database (CRD42020152489). The following guiding question was considered: What is the effect of nutritional interventions, combined or not with the practice of physical activity, on the adiposity of the newborns of pregnant women with overweight or obesity?

### 2.1. Eligibility Criteria

#### 2.1.1. Type of Study

Original articles covering lifestyle intervention studies in pregnant women with overweight or obesity and the effect on neonatal adiposity, published in English, Portuguese or Spanish, published up to 28 October 2019, were considered eligible. Non-randomized clinical trials and quasi-experimental studies, prospective or retrospective observational cohort studies, cross-sectional studies, cases and case series, and experimental studies involving animals were not considered eligible for inclusion, as well as unpublished articles, abstracts or dissertations and theses.

#### 2.1.2. Study Population

Pregnant women ≥18 years of age, preconception BMI ≥ 25 kg/m^2^, singleton pregnancy, gestational age at recruitment up to 20 weeks, follow-up period up to 36 weeks and 6 days. Studies that included women with type 1 or type 2 diabetes prior to pregnancy or that had an early diagnosis of gestational diabetes prior to recruitment were excluded.

#### 2.1.3. Intervention

Nutritional interventions based on dietary guidelines combined or not with the incentive of physical activity were considered. Further details about the design and the intervention were obtained from all included studies.

#### 2.1.4. Outcomes

##### Primary Outcome

The direct or indirect measurement of the infants’ body fat was considered, expressed in absolute (mass) or relative (percentage) values, performed up to 28 days after the birth. Fat mass and fat-free mass are considered to be more sensitive markers of intrauterine growth compared to indirect measures of adiposity such as body mass index and absolute values of anthropometric measures [29]. Furthermore, the neonatal period corresponds to fetal exposure to prenatal growth factors and the first peak of body fat accumulation [30].

In the qualitative analysis of the studies included, possible confounding factors were considered in the expression of the results. Additionally, in the quantitative analysis, it was verified whether there was a change in the effect on the outcome according to the method of measuring adiposity.

##### Secondary Outcomes

Secondary outcomes considered were birth weight; neonatal fat-free mass, expressed as mass or relative percentage; and maternal gestational weight gain.

### 2.2. Information Sources and Search

The databases PubMed (MEDLINE), Embase, Web of Science, Scopus and the Latin American and Caribbean Literature in Health Sciences (LILACS) were searched to identify eligible articles published up to 28 October 2019. The options “Advance Search” and “All fields” were selected: (((((Pregnancy OR Pregnancies OR Gestation OR Gravidity OR Pregnant) AND (Overweight OR Obesity OR Obese)))) AND ((Diet OR Diets OR Dietary OR Nutrition OR “Prenatal Nutritional Physiological Phenomena” OR “Life Style” OR “Life Styles” OR Lifestyle OR Lifestyles))) AND (((Adiposity OR “Body Composition” OR “Body Compositions”) AND (“Infant, Newborn” OR Newborns OR Newborn OR Neonate OR Neonates OR Offspring))). The search strategy adapted for each database is available in full as Supplementary File S1.

### 2.3. Study Selection

The Rayyan^®^ reference manager software (Qatar Computing Research Institute, HBKU, Doha, Qatar, v.1.0) [31] was used for the storage and management of the publications. After eliminating duplicate articles, the titles and abstracts were read by two researchers independently (Naiara Franco Baroni and Nayara Ragi Baldoni), minimizing possible bias in the selection and exclusion of the studies. In this stage, contact was made via e-mail with the authors to formally request the publications in full [32,33]. In the eligibility stage, a third researcher (Lívia Castro Crivellenti.) assessed the possible conflicts and, by consensus, the articles to be included in the review were defined. Finally, the list of references of the included articles was consulted as an additional source of eligible studies.

### 2.4. Data Extraction

Data extraction was performed separately by two researchers (Naiara Franco Baroni and Nayara Ragi Baldoni) using a standardized form relating the coding of variables that were organized in the respective Excel spreadsheets, namely: identification data (title, authors, date of publication and place of investigation); study model, sample size and characteristics of the study population (preconception BMI, age, ethnicity and gestational age at delivery); details of the intervention (type, method, strategy, professional that performed it, frequency and period of follow-up in gestational weeks); primary outcome assessment method (neonatal adiposity); birth weight; neonatal fat-free mass; and gestational weight gain.

### 2.5. Risk of Bias Assessment

The risk of bias in each study was assessed in duplicate (Naiara Franco Baroni and Nayara Ragi Baldoni.) with the aid of a standardized form [34] and the RevMan^®^ software (Copenhagen, Denmark) v5.3 [35]. The Cochrane Handbook for Systematic Reviews of Interventions tool [Risk of bias 2 (RoB 2)] [28] was used as a reference.

### 2.6. Synthesis of the Data

After reading and including the studies, the methods used were evaluated and the data were extracted and synthesized from the meta-analysis. The meta-analysis was calculated using the inverse variance for continuous data expressed as mean difference (MD), using the random effect model with a 95% confidence interval (CI). For the analysis of the main outcome, data were also considered according to the measurement method. The quantitative synthesis was performed using the RevMan^®^ software v5.3 [35]. The heterogeneity or inconsistency of the studies, estimated through I², of up to 50% were considered [28]. Secondary outcomes were assessed and presented as Supplementary Materials.

The outcomes were submitted to Grading of Recommendations Assessment, Development and Evaluation (GRADE) [36], considering five factors (reporting bias, inconsistency, indirectness of evidence, imprecision and other considerations such as publication bias) to assess the quality of the body of evidence, using the GRADE Profiler^®^ software Hamilton, ON, Canada, v1 [37]. Decisions to decrease confidence in effect estimates were justified using footnotes and comments of importance for each outcome assessed. The evaluation of heterogeneity and risk of bias was used, with the evidence quality only lowered if risk of bias was detected.

## 3. Results

### 3.1. Search Results

**Figure 1:**
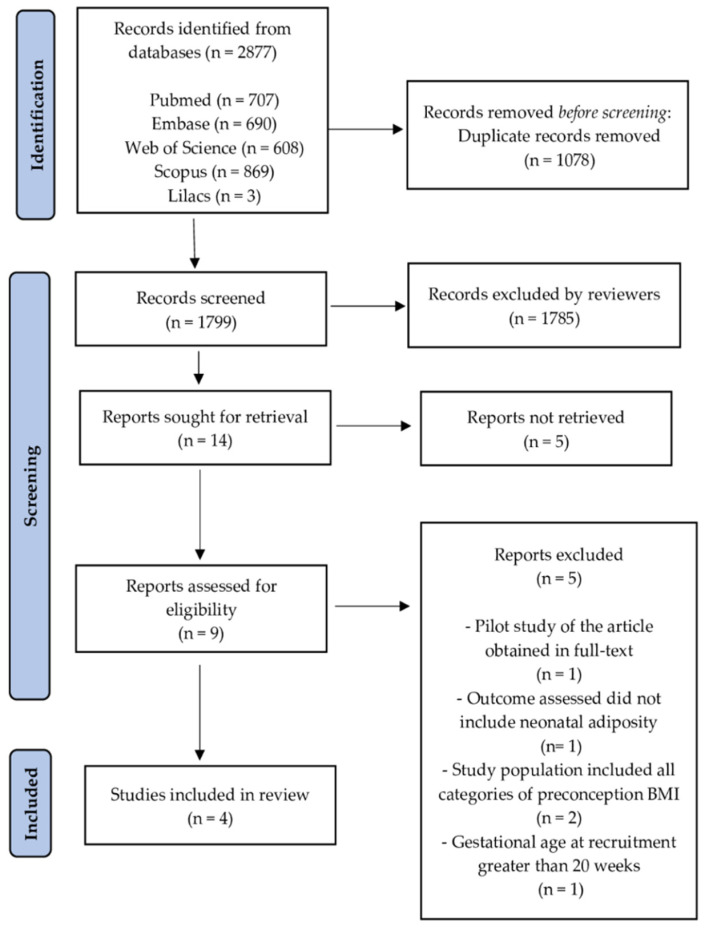
Preferred Reporting Items for Systematic Reviews and Meta-Analyses (PRISMA) 2020 flow diagram: identification and selection of studies process. BMI: body mass index.

All publications identified were in the English language. One thousand seven hundred ninety-nine publications were screened after removing duplicates. The selection of studies consisted of reading the titles and abstracts, with a total of 14 studies screened (Reports sought for retrieval). Of these, nine were eligible (Reports assessed for eligibility). After reading them in full, five were excluded. In total, four studies were included in the qualitative and quantitative synthesis [37,38,39,40]. The process of identification and selection of articles included is shown in Figure 1.

### 3.2. Characteristics of the Studies

All studies included were conducted in developed countries: Dodd et al. [38] in Australia; Gallagher et al. [39] and van Horn et al. [40] in the USA; and van Poppel et al. [41], a multicentric study, in the United Kingdom, Ireland, Netherlands, Austria, Poland, Italy, Spain, Denmark and Belgium. Of these, three used a 1:1 proportion allocation between the treatment groups [38,39,40], and one employed a 3:1 proportion [41]. The articles were published between 2016 and 2019. Table 1 summarizes the characteristics of the included studies.

Dodd et al. [38], Gallagher et al. [39] and van Horn et al. [40] included pregnant women with overweight or obesity in the study population (preconception BMI ≥ 25 kg/m^2^). In the study by van Poppel et al. [41] only pregnant women with obesity (preconception BMI ≥ 30 kg/m^2^) were included. The mean age of pregnant women at the baseline ranged from 24.2–37.8 years in the intervention group (IG) and 24.6–38.5 years in the control group (CG). The terms to designate ethnicity varied, however, it was possible to suggest a higher frequency of white women (IG 46 to 90% CG 48 to 91.3%).

Among the studies included, interventions based on dietary guidelines combined with regular practice of physical activity were considered, mentioned as lifestyle interventions in this review. In the study by van Horn et al. [40], the content of the intervention also included guidance on increasing sleep time. All clinical trials included applied strategies that aimed to stimulate the empowerment and autonomy of the pregnant women to achieve the goals and were monitored by the researchers.

Two studies used individualized interventions associated with the incentive to participate in groups: Gallagher et al. [39] and van Horn et al. [40], derived from the Lifestyle Intervention for Two (LIFT) study and the Maternal-Offspring Metabolics: Family Intervention Trial (MOMFIT) study, respectively.

Two other studies carried out individualized interventions with remote monitoring: Dodd et al. [38] and van Poppel et al. [41], derived from the Limiting weight gain during pregnancy (LIMIT Study) and Vitamin D and Lifestyle Intervention for Gestational Diabetes Mellitus Prevention (DALI Study), respectively. It should be noted that all studies included adopted appropriate gestational weight gain as the primary outcome.

In three studies [38,39,40], the pregnant women in the intervention group received an individualized food plan with lifestyle change goals determined from healthy eating guidelines. A nutritionist was present in at least one session during the follow-up period. While in one of the studies [41] the pregnant women in the intervention group received lifestyle counseling through key messages applied by a coach. In all the studies included, the first intervention session was conducted at the beginning of the second gestational trimester, however, there was no information about the mean gestational age at the time. Only two studies discussed adherence to the intervention [39,40]. The treatment of pregnant women in the control group consisted of the usual prenatal care of the health services [38,41] only, or complemented with a general approach of the research team on healthy habits during pregnancy without the establishment of weight gain goals [39,40].

Two methods of measuring neonatal adiposity were identified: infant air displacement plethysmography (ADP) estimated by the PEA POD^®^ system (COSMED USA, Inc., Concord, CA, USA) used by Gallagher et al. [39] and van Horn et al. [40] (*n* = 389); and the anthropometric model for estimating children’s body composition proposed by Deierlein et al. [42] adopted by Dodd et al. [38] and van Poppel et al. [41] (*n* = 1105).

Additional outcomes of birth weight (*n* = 607) and gestational weight gain (*n* = 651) were assessed by Gallagher et al. [39], van Horn et al. [40] and van Poppel et al. [41]; a third additional outcome was the newborn’s fat-free mass (*n* = 1296) assessed by Dodd et al. [38], Gallagher et al. [39] and van Poppel et al. [41].

### 3.3. Risk of Bias Assessment

The overall risk assessment consisted of a high risk of bias. Five domains were evaluated in each study (Table 2): Randomization process: “Some concerns” were attributed to one study [39], because not enough information was reported. Deviations from the intended intervention: “Some concerns” were attributed to one study [38], because no details on blinding were reported. Missing outcome data: Considering per-protocol analysis, a “low” risk of bias was attributed to all studies. Measurement of the outcome: Some concerns were attributed to the studies [38,41], because they did not explain the blinding in the outcome assessment. It should be highlighted that two studies [39,40] were evaluated as “high” risk of bias, because they did not consider smoking or exposure to tobacco during pregnancy as an adjustment factor to statistically determine the results. Selection of the reported results: All studies were attributed a “low” risk of bias [38,39,40,41].

### 3.4. Effect of the Interventions

**Figure 2:**
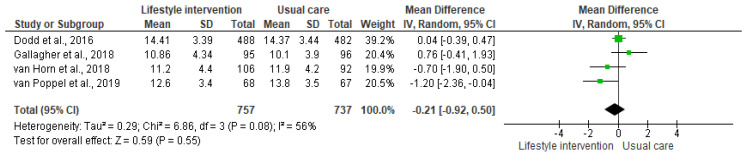
Effect of the lifestyle interventions in pregnant women with excessive body weight on neonatal adiposity [38,39,40,41]. SD = standard deviation; CI = confidence interval; Tau^2^ = Tau-squared test; Chi^2^ = Chi-squared test; Df = difference; P = *p* value; I^2^ = heterogeneity; Z = Z test.

**Figure 3:**
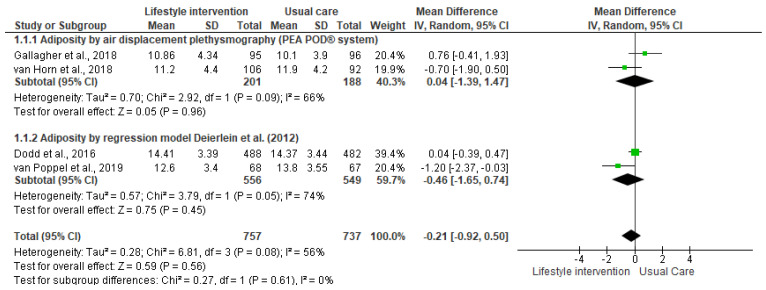
Effect of the lifestyle interventions in pregnant women with excessive body weight on neonatal adiposity according to the assessment method [38,39,40,41]. SD = standard deviation; CI = confidence interval; Tau^2^ = Tau-squared test; Chi^2^ = Chi-squared test; Df = difference; P = *p* value; I^2^ = heterogeneity; Z = Z test.

The lifestyle interventions with pregnant women had no effect on neonatal adiposity [I^2^ = 56%, *n* = 1494, MD = −0.21, CI = (−0.92, 0.50)]. Although the results suggest a positive effect trend, since the percentage of body adiposity was lower in the newborns in the intervention groups, the differences compared to the control groups were not significant (Figure 2). The analysis stratified by the outcome measurement method indicated the same result (Figure 3). Accordingly, the results of the meta-analyses are equal with a moderate degree of heterogeneity.

The analysis of secondary outcomes showed a positive effect of the intervention in reducing gestational weight gain considering the mean difference between the treatment groups [I^2^ = 0%, *n* = 651, MD = −1.97 CI = (−2.7, −1.24)] (Supplementary Figure S1) however, no effect of the intervention was observed regarding the neonatal outcomes of birth weight [I^2^ = 55%, *n* = 607, MD = 23.26, CI = (−102.73, 149.24)] (Supplementary Figure S2) and the fat-free mass [I^2^ = 50%, *n* = 1296, MD = −0.68, CI = (−72.87, 71.51)] (Supplementary Figure S3).

The GRADE evaluation indicated low confidence in the evidence regarding the effect of the intervention on neonatal adiposity, with a very serious risk of bias. However, moderate confidence was observed in relation to the secondary outcomes, with a serious risk of bias (Supplementary Figure S4).

## 4. Discussion

The present systematic review study with meta-analysis evaluated randomized clinical trials of lifestyle interventions in pregnant women with overweight or obesity, and the effects on neonatal adiposity. Four studies were included in the analyses, all conducted in developed countries with similar population characteristics and performance scenarios. There was no effect of the interventions on neonatal adiposity with low confidence, according to GRADE evaluation. It is possible that new studies could modify the results found.

Previous systematic reviews of interventions in the first 1000 days of life, between conception and 24 months, have evaluated excessive body weight as the outcome among children and adolescents aged six months to 18 years. These studies showed a high degree of heterogeneity due to the variability of the types of intervention and methods of anthropometric measurements [43,44]. Therefore, there is no evidence regarding the influence of interventions during pregnancy and the prevention of obesity in children. However, the researchers stress the importance of future studies operating at a systemic and environmental level to prevent childhood obesity.

A systematic review of observational studies suggested that exposure at the first 1000 days of life to preconception maternal BMI, excessive gestational weight gain, prenatal tobacco exposure, high birth weight, and accelerated infant weight gain are relevant risk factors for obesity in late childhood [45]. Important to note that two studies included in the present review [39,40] did not consider smoking or exposure to tobacco as a confounder in the analyses. In addition, the secondary outcomes of birth weight and fat-free mass did not differ between the treatment groups, with low confidence in the results. However, there was less weight gain among the pregnant women in the intervention group when compared to the control group, with moderate confidence in the results. However, it is not possible to affirm that it was clinically significant since the mean weight gain between the groups was considered, and not the proportion of women with adequate weight gain according to the Institute of Medicine (IOM) recommendation [46].

A systematic review and meta-analysis of observational studies assessed the dose-response association between maternal preconception BMI and childhood obesity in children aged between one and 18 years. The evidence showed significantly increased chances of childhood obesity with an increase in maternal BMI, where the association was stronger among women with preconception obesity than among those that were overweight prior to conception, 264% and 89%, respectively [47].

A meta-analysis of clinical trials of lifestyle interventions with overweight or obese pregnant women showed that even with a significant reduction in weight gain in the intervention group, no impact was found on other outcomes considered as risks for childhood obesity, including birth weight [48]. Preconception BMI ≥ 30 kg/m^2^ may be more relevant than excessive weight gain during pregnancy, as it favors greater neonatal adiposity and, in the long term, childhood obesity and the emergence of chronic non-communicable diseases. In light of epigenetics, this is due to an inverse relationship between the increase in preconception BMI and insulin sensitivity, which allows the fetus a greater availability of energy nutrients, causing a greater impact on neonatal outcomes [49,50,51,52,53]. Accordingly, using mixed categories of BMI in the same population may have interfered with the outcome results of the present review.

It is considered that initiating interventions prior to conception in women of reproductive age who have obesity is the most appropriate strategy for metabolic control before, during, and after pregnancy. With this, there is a greater chance of breaking with the intergenerational cycle of obesity, enabling better maternal and fetal health outcomes in the short and long terms [54,55,56,57,58,59]. In addition, for greater effectiveness of the interventions, the adoption is recommended of expanded approaches beyond the health sector, applied to the ethnic-socio-cultural context of different populations [60,61,62,63].

The global epidemic of obesity has led to an increase in women of reproductive age with obesity. The maternal and child double burden of malnutrition in low-income and middle-income countries (LMICs) encompasses both undernutrition and a growing problem with overweight and obesity [64]. Excessive body weight is frequent among pregnant women from LMICs in Europe, the eastern Mediterranean region, and the Americas. In Africa, obesity rates among pregnant women ranged from 0.7% to 26.8% [65]. It is assumed that using mixed categories of BMI in the study population may have interfered with the findings of this systematic review. In addition, the characteristics of the population and study scenarios may have influenced the results, with studies conducted in low and medium development countries and at other levels of health care possibly producing different results from those observed.

### Methods of Measuring the Newborn’s Body Composition

Two methods of measuring for estimative the newborn’s body composition were identified: air displacement plethysmography (ADP, PEA POD^®^ system), used by Gallagher et al. [39] and van Horn et al. [40], and the anthropometric model proposed by Deierlein et al. [42], adopted by Dodd et al. [38] and van Poppel et al. [41]. The meta-analysis stratified by the measurement method also did not indicate any effect of the intervention on neonatal adiposity.

There are many methods available to assess body composition at birth and all present limitations. The ADP is a densitometry technique. By definition, the density of the whole body is body mass divided by body volume. ADP measurement procedures are performed in air, compressed under isothermal conditions, using gas Boyle’s Law and Poisson’s Law to determine body volume. Body density is then used in a two-compartment model, fixed density of fat (0.9007 g/mL), to calculate the percentage of fat, fat mass, and fat-free mass, age, and sex-specific density values. It is considered the gold standard, that is, the reference method, with its main advantage being high accuracy. However, it also has a high cost [66,67,68,69,70,71].

Deierlein et al. [42] determined an anthropometric model, using backward stepwise linear regression, including infant gender/sex, age, race/ethnicity, weight, and skinfold thickness measurements showing 81% agreement in the estimates in relation to the PEA POD^®^ system (ADP) in a multi-ethnic population of term infants at 1–3 days post-delivery. The final statistical model that predicted neonatal fat mass (kg) was: (−0.012 − 0.064 × gender + 0.024 × day of measurement post-delivery − 0.150 × weight (kg) + 0.055 × weight (kg)2 + 0.046 × ethnicity + 0.020 × sum of three skinfold thicknesses (triceps, subscapular, and thigh)).

The authors highlighted the following limitations of the model: it estimates only the amount of subcutaneous adipose tissue in the central and peripheral regions, not being possible to obtain information on the amount of visceral fat, and has a limited ability to predict the amount of fat mass in small and large for gestational age newborns. On the other hand, it is the only anthropometric model for estimating the newborn’s body composition that considers ethnicity as one of the predictor variables. It should be emphasized that, regarding the genetic aspect, ethnicity can influence the amount of fat-free mass of the newborn, and the ratio between fat mass and fat-free mass of body fat at birth is an important characteristic to assess potential metabolic risks in childhood and throughout life [72].

In general, anthropometric models has low accuracy, however, greater applicability and low cost, being the most feasible in population studies. Although the need for an alternative to ADP is recognized, the performance of the predictive model compared to this technology is negatively evaluated, as it can overestimate the fat mass values in the newborn [73,74].

When adopting the predictive model, Dodd et al. [38] clearly reported the time elapsed between the birth and the measurements. In addition, they calculated the intra-observer variation (0.55–0.88), which can be considered a way to reduce the method’s limitation regarding possible variability. The study by van Poppel et al. [41] was the only one to present any significant effect of the intervention on neonatal adiposity, since the neonates in the intervention group had a lower percentage of body fat mass compared to the newborns in the control group. However, it was observed that no technique was adopted to reduce the limitations of the method in a population of unrepresentative size.

The use of predictive models to estimate neonatal adiposity in population studies is considered valid, emphasizing the need to apply techniques that reduce their limitations. Therefore, to advance the understanding of how early life exposures affect obesity and metabolic risk, improvement in the accuracy and standardization of technologies to measure infant body composition are greatly needed [75].

## 5. Potentials and Limitations

Limitations at the level of the studies were characterized regarding the performance scenario, all conducted in developed countries. In addition, the use of mixed BMI categories in the study population made it impossible to analyze by subgroup. The use of different methods for measuring neonatal adiposity was considered a limitation related to the outcome, as it may have interfered with the findings. Accordingly, to minimize this limitation, it was decided to present the meta-analysis stratified by the method of measuring body composition. Moreover, it is possible that new studies could modify the results found since the search was conducted at the end of 2019.

The development of a comprehensive search strategy and the use of the PRISMA and Cochrane review guidelines were considered strengths of this review. However, the period elapsed after the last search carried out in the databases and the non-inclusion of gray literature as a source of information, especially the registration databases of clinical trial protocols, characterizing a limitation related to the risk of publication bias.

## 6. Conclusions

It was concluded that there is no evidence for the effect of the lifestyle interventions in pregnant women with overweight or obesity on neonatal adiposity, fat-free mass, and birth weight. While there was a positive effect of the intervention on the mean gestational weight gain in the study population. Further studies are needed, especially in countries of low and medium socioeconomic development with different ethnic-racial populations, aimed at encouraging the consumption of a healthy diet and the regular practice of physical activity in women of reproductive age before, during, and after pregnancy. Studies should present the outcome according to the category of preconception BMI, avoiding confusion bias. New studies could contribute to the definition of effective interventions in the prevention of childhood obesity and, consequently, support public health actions.

## Figures and Tables

**Table 1:** Characteristics of the included studies.

Study ID, Country	Methods	Population	Intervention	Main Outcome
**Dodd et al., 2016 [38]** LIMIT study, Australia	Model: Randomized clinical trial 1:1 stratified by parity, BMI and maternity Scenario: Three largest maternity hospitals in Adelaide Follow-up time: Start: 10–20 GW End: 36 GW	*n* total of randomized pregnant women: 2212 *n* by BMI categories of the groups at baseline: (I) *n* = 488 Overweight = 187 (38.32%) Obesity = 301 (61.68%) (C) *n* = 482 Overweight = 200 (41.49%) Obesity = 282 (58.51%) Median BMI of the groups: (I) 31.3 kg/m^2^ (28.2–35.9) (C) 31.2 kg/m^2^ (27.8–36.6)	Type and strategy: (I): Lifestyle: individualized dietary guidance using the Australian Food Guides standards + incentive to practice regular walking; motivational stimulus to exercise autonomy in monitoring the goals Frequency 3 face-to-face meetings (12 to 22 GW, 28 GW, 36 GW) + 3 phone calls (22 GW, 24 GW, 32 GW) (C): Usual prenatal care	Effect of dietary and lifestyle counseling during prenatal care on anthropometric measurements of the newborn % adherence = 44.05% (I) *n* = 488 (C) *n* = 482 Predictive method of estimating neonatal adiposity by regression model; measurements performed up to 72 h after birth Fat mass (g) (I) 522.72 (± 180.7) (C) 523.48 (± 189.05) (adjusted *p* value *=* 0.94) Fat-free mass (g) (I) 3026.64 (± 339.96) (C) 3030.07 (± 362.54) (adjusted *p* value = 0.97)
**Gallagher et al., 2018 [39]** LIFT study, USA	Model: Randomized clinical trial 1:1 Scenario: Private hospitals and clinics in New York Follow-up time: Start: 9–15 GW6d End: 35–36 GW6d	*n* total of randomized pregnant women: 210 Frequency by BMI categories of the groups at baseline: (I) *n* = 105 Overweight = 65 (62%) Obesity = 40 (38%) (C) *n* = 105 Overweight = 60 (57%) Obesity = 45 (43%) Mean BMI (±SD) of the groups: (I) 30.1 (4.1) (C) 30.7 (5.0)	Type and strategy: (I) Lifestyle: individualized guidance on diet modification using Diabetes care guidelines adapted to control gestational weight gain + incentive to practice physical activity + stimulation to modify behavior and social support strategies: Frequency: Face-to-face meetings: 1 introductory session + one meeting every 15 days. Weekly contacts by phone/email. Group meetings every 8 weeks (C): Usual prenatal care + introductory approach to nutritional care during pregnancy + group meetings on health during pregnancy not contemplating caloric restriction	Effectiveness of control of gestational weight gain in the 2nd and 3rd trimester on the newborn’s body composition % adherence = 90.95% (I) *n* = 95 (C) *n* = 96 Direct method: infant ADP; measurements carried out between the 1st and 4th day for full term babies or up to 36 weeks after the last maternal menstruation date for preterm babies Fat mass (g) (I) 360 (± 173) (C) 324 (± 157) (adjusted *p* value = 0.08) Fat-free mass (g) (I) 2871 (± 404) (C) 2786 (± 405) (adjusted *p* value = 0.03)
**van Horn et al., 2018 [40]** MOMFIT study, USA	Model: Randomized clinical trial 1:1 Scenario: Northwestern Memorial Hospital and medical clinics in Chicago Follow-up time: Start: 16 GW End: 35–36 GW6d	*n* total of randomized pregnant women: 281 Frequency by BMI categories of the groups at baseline: (I) *n* = 140 Overweight = 63 (45.0%) Obesity = 77 (55.0%) (C) *n* = 141 Overweight = 64 (45.4%) Obesity = 77 (54.6%) Preconception BMI mean (±SD) of the groups: (I) 31.0 (4.0) (C) 31.0 (4.0)	Type and strategy: (I) Lifestyle: individualized guidance on diet modification using the MAMA-DASH standard (adapted Dietary Approach to Stop Hypertension) + incentive to practice physical activity + encouragement of autonomy, following the Motivational Interview principles Frequency 3 face-to-face meetings (15 GW, 23 GW and 33 GW) Contact through the “LOSEIT!” app. emails, text messages, electronic brochures and telephone calls. Group sessions during the 1st and 2nd gestational trimester (C): American Physical Activity Guidelines and the Recommendations of the American College of Gynecology and Obstetrics, access to websites and guidelines of National Organizations and authorities on nutrition, physical activity and healthy pregnancy	Secondary outcomes include anthropometric measurements at birth, however, do not mention neonatal adiposity % adherence = 70.5% (I) *n* = 106 (C) *n* = 92 Direct method: infant ADP; measurements carried out between 24 and 72 h of life The intervention had no effect on adiposity and neonatal body composition in relation to the control group. Body fat percentage: (I) 11.2 (± 4.4) (C) 11.9 (± 4.2) (adjusted *p* value = 0.56) * did not present mass values
**van Poppel et al., 2019 [41]** DALI study, United Kingdom, Ireland, Netherlands, Austria, Poland, Italy, Spain, Denmark and Belgium	Model: Randomized clinical trial 3:1 Scenario: Prenatal clinics and maternity hospitals in nine European countries Follow-up time: Start: ˂20 GW End: 35–37 GW	*n* total of randomized pregnant women: 326 Preconception BMI mean (±SD) of the groups at baseline (I_1_): *n* = 92 pre BMI: 34.2 ± 4.6 kg/m^2^ (I_2_): *n* = 80 pre BMI: 33.6 ± 3.6 kg/m^2^ (C): *n* = 80 pre BMI: 33.7 ± 3.7 kg/m^2^ (*) Difference between the total number of randomized pregnant women and the intervention group that received only physical activity guidelines, which was not considered in this study.	Type and strategy: (I): Lifestyle: Dietary counseling through individual sessions conducted by a coach trained to deliver 7 key messages related to the quality of food, portion size and thus caloric restriction + 5 messages to encourage the practice of aerobic and resistance physical activity * The content of the strategy was based on a previous study focusing on the prevention of Diabetes Mellitus in adults * Up to 5 kg weight gain limit established. Frequency: 5 face-to-face meetings (4 held between 24–28 GW and completed by 35 GW) + 4 contacts by phone or email (C): Usual prenatal care	Effect of intervention on neonatal anthropometry and umbilical cord leptin, and profile of neonatal adiposity % adherence = 62.6% (I_1_): *n* = 69 (I_2_): *n* = 68 (C): *n* = 67 Predictive method of estimating neonatal adiposity by regression model Fat mass (g) (I_1_) 492 (± 213) (adjusted *p* value *=* 0.45) (I_2_) 451 (± 171) (adjusted *p* value *=* 0.04) (C) 511 (181) Fat-free mass (g) (I_1_) 3105 (± 410) (adjusted *p* value *=* 0.98) (I_2_) 3029 (± 351) (adjusted *p* value *=* 0.20) (C) 3111 (± 339)

Note: LIMIT study = Limiting weight gain during pregnancy; LIFT study = Lifestyle Intervention for Two; MOMFIT study = Maternal Offspring Metabolics: Family Intervention Trial; DALI study = Vitamin D and Lifestyle Intervention for Gestational Diabetes Mellitus Prevention; (C) = control group; (I) = intervention group; (I_1_) = diet intervention group; (I_2_) = lifestyle intervention group: diet + physical activity; d = days; SD = standard deviation; *n* = number; ADP = infant air displacement plethysmography system; GW = gestational weeks.

**Table 2:** Sources of bias and risk classification in each included study.

Domain	Risk Classification of Studies			
	Dodd et al. (2016) [38]	Gallagher et al. (2018) [39]	van Horn et al. (2018) [40]	van Poppel et al. (2019) [41]
Randomization process	Low	Some concerns	Low	Low
Deviations from intended interventions	Some concerns	Low	Low	Low
Missing outcome data	Low	Low	Low	Low
Measurement of the outcome	Some concerns	High	High	Some concerns
Selection of the reported result	Low	Low	Low	Low

## Data Availability

Datasets arising from the study might be available upon reasonable request from the corresponding author Daniela S. Sartorelli.

## Supplementary Materials

The following are available online at https://www.mdpi.com/article/10.3390/nu13061903/s1. Supplementary File S1: Search strategy generated in the databases from the terms and between constituent terms of PICO. Table S1: PRISMA 2020 Checklist. Table S2: PRISMA Abstract Checklist. Figure S1: Effect of the lifestyle intervention with overweight or obese pregnant women on gestational weight gain; Figure S2: Effect of the lifestyle interventions in pregnant women with excessive body weight on birth weight; Figure S3: Effect of the lifestyle interventions in pregnant women with excessive body weight on the fat-free mass of the newborn; Figure S4: Summary of findings: The effect of the lifestyle intervention with overweight/obese pregnant women in relation to the neonatal adiposity.
