# Supplementary material for: Do Lifestyle Interventions in Pregnant Women with Overweight or Obesity Have an Effect on Neonatal Adiposity? A Systematic Review with Meta-Analysis
Source: Nutrients. 2021 Jun 1;13(6):1903. doi: 10.3390/nu13061903 (PMC8228378; doi:10.3390/nu13061903)
Supplement: Supplementary file 1 [file nutrients-13-01903-s001.zip › supplementary/Supplementary 1_ Search strategy.pdf]

Supplementary File 1. Search strategy generated in the databases from the terms and between constituent terms of PICO.

PUBMED (28 October 2019)

(P) Population
((Pregnancy OR Pregnancies OR Gestation* OR Gravidity OR Pregnant) AND (Overweight OR Obesity OR Obese))

(I) Intervention
(Diet OR Diets OR Dietary OR Nutrition OR "Prenatal Nutritional Physiological Phenomena" OR "Life Style" OR "Life Styles" OR Lifestyle OR Lifestyles)

(O) Outcome
((Adiposity OR "Body Composition" OR "Body Compositions") AND ("Infant, Newborn" OR Newborns OR Newborn OR Neonate OR Neonates OR Offspring))

n = 707
(((((Pregnancy OR Pregnancies OR Gestation* OR Gravidity OR Pregnant) AND (Overweight OR Obesity OR Obese)))) AND ((Diet OR Diets OR Dietary OR Nutrition OR "Prenatal Nutritional Physiological Phenomena" OR "Life Style" OR "Life Styles" OR Lifestyle OR Lifestyles))) AND (((Adiposity OR "Body Composition" OR "Body Compositions") AND ("Infant, Newborn" OR Newborns OR Newborn OR Neonate OR Neonates OR Offspring)))

EMBASE (28 October 2019)

(P) Population
(Pregnancy OR Gestation* OR Gravidity OR Pregnant) AND (Obesity OR Overweight OR Obese)

(I) Intervention
Diet OR Nutrition OR Dietary OR Lifestyle OR “Life Style”

(O) Outcome
(“Body Composition” OR “Composition, Body” OR Adiposity) AND (Newborn OR “Infant, Newborn” OR Infant OR Neonate OR Progeny OR Offspring)

n = 690
(**“pregnancy”**/exp OR **pregnancy** OR **gestation*** OR **“gravidity”**/exp OR **gravidity** OR **pregnant**) AND (**“obesity”**/exp OR **obesity** OR **“overweight”**/exp OR **overweight** OR **obese**) AND **“diet”**/exp OR **diet** OR **“nutrition”**/exp OR **nutrition** OR **dietary** OR **“lifestyle”**/exp OR **lifestyle** OR “**life style”**/exp OR “**life style” AND** (**“body composition”**/exp OR **“body composition”** OR **“composition, body”**/exp OR **“composition, body”** OR **adiposity**) AND (**“newborn”**/exp OR **newborn** OR “**infant, newborn”**/exp OR **“infant, newborn”** OR **“infant”**/exp OR **infant** OR **“neonate”**/exp OR **neonate** OR **“progeny”**/exp OR **progeny** OR **“offspring”**/exp OR **offspring**) #4 AND [embase]/lim NOT ([embase]/lim AND [medline]/lim)

WEB OF SCIENCE (28 October 2019)

(P) Population

((Pregnancy OR Pregnancies OR Gestation* OR Gravidity OR Pregnant) AND (Overweight OR Obesity OR Obese))

(I) Intervention
(Diet OR Diets OR Dietary OR Nutrition OR "Prenatal Nutritional Physiological Phenomena" OR "Life Style" OR "Life Styles" OR Lifestyle OR Lifestyles)

(O) Outcome
((Adiposity OR "Body Composition" OR "Body Compositions") AND ("Infant, Newborn" OR Newborns OR Newborn OR Neonate OR Neonates OR Offspring))

n = 608
TS = (((Pregnancy OR Pregnancies OR Gestation* OR Gravidity OR Pregnant) AND (Overweight OR Obesity OR Obese)) AND (Diet OR Diets OR Dietary OR Nutrition OR "Prenatal Nutritional Physiological Phenomena" OR "Life Style" OR "Life Styles" OR Lifestyle OR Lifestyles) AND ((Adiposity OR "Body Composition" OR "Body Compositions") AND ("Infant, Newborn" OR Newborns OR Newborn OR Neonate OR Neonates OR Offspring)))
Índices=SCI-EXPANDED, SSCI, A&HCI, CPCI-S, CPCI-SSH, ESCI Tempo estipulado=Todos os anos

SCOPUS (28 October 2019)

(P) Population
((Pregnancy OR Pregnancies OR Gestation* OR Gravidity OR Pregnant) AND (Overweight OR Obesity OR Obese))

(I) Intervention
(Diet OR Diets OR Dietary OR Nutrition OR "Prenatal Nutritional Physiological Phenomena" OR "Life Style" OR "Life Styles" OR Lifestyle OR Lifestyles)

(O) Outcome
((Adiposity OR "Body Composition" OR "Body Compositions") AND ("Infant, Newborn" OR Newborns OR Newborn OR Neonate OR Neonates OR Offspring))

n = 869
(TITLE-ABS-KEY (((pregnancy OR pregnancies OR gestation* OR gravidity OR pregnant) AND (overweight OR obesity OR obese))) AND TITLE-ABS-KEY ((diet OR diets OR dietary OR nutrition OR "Prenatal Nutritional Physiological Phenomena" OR "Life Style" OR "Life Styles" OR lifestyle OR lifestyles)) AND TITLE-ABS-KEY (((adiposity OR "Body Composition" OR "Body Compositions") AND ("Infant, Newborn" OR newborns OR newborn OR neonate OR neonates OR offspring)))) AND (LIMIT-TO (DOCTYPE, "ar") OR LIMIT-TO (DOCTYPE, "re") OR LIMIT-TO (DOCTYPE, "cp"))
Filter: Document type: artigos, revisão e conference paper

LILACS (28 October 2019)

(P) Population
((Pregnancy OR Embarazo OR Gravidez OR Gestação OR "Pregnant Women" OR "Mujeres Embarazadas" OR Gestantes OR Grávida OR "Mulheres Grávidas") AND (Overweight OR Sobrepeso OR Obesity OR Obesidad OR Obesidade))

(I) Intervention
(Diet OR Dieta OR "Regime Alimentar" OR "Estilo de Vida" OR "Life Style")

(O) OUTCOME
((Adiposity OR Adiposidad OR Adiposidade OR "Body Composition" OR "Composición Corporal" OR "Composição Corporal") AND ("Infant, Newborn" OR "Recién Nacido" OR "Recém-Nascido" OR "Criança Recém-Nascida" OR "Crianças Recém-Nascidas" OR Neonato OR Neonatos OR "Recém-Nascidos"))

$n = 3$
tw:((tw:(((pregnancy OR embarazo OR gravidez OR gestação OR "Pregnant Women" OR "Mujeres Embarazadas" OR gestantes OR grávida OR "Mulheres Grávidas") AND (overweight OR sobrepeso OR obesity OR obesidad OR obesidade)))) AND (tw:((diet OR dieta OR "Regime Alimentar" OR "Estilo de Vida" OR "Life Style"))) AND (tw:(((adiposity OR adiposidad OR adiposidade OR "Body Composition" OR "Composición Corporal" OR "Composição Corporal") AND ("Infant, Newborn" OR "Recién Nacido" OR "Recém-Nascido" OR "Criança Recém-Nascida" OR "Crianças Recém-Nascidas" OR neonato OR neonatos OR "Recém-Nascidos")))))
