# Supplementary material for: Do Lifestyle Interventions in Pregnant Women with Overweight or Obesity Have an Effect on Neonatal Adiposity? A Systematic Review with Meta-Analysis
Source: Nutrients. 2021 Jun 1;13(6):1903. doi: 10.3390/nu13061903 (PMC8228378; doi:10.3390/nu13061903)
Supplement: Supplementary file 1 [file nutrients-13-01903-s001.zip › supplementary/Supplementary 3_ PRISMA Abstract Checklist.pdf]

Supplementary Table S2. PRISMA Abstract Checklist.

Topic	No.	Item	Reported?
TITLE			
Title	1	Identify the report as a systematic review.	Yes
BACKGROUND			
Objectives	2	Provide an explicit statement of the main objective(s) or question(s) the review addresses.	Yes
METHODS			
Eligibility criteria	3	Specify the inclusion and exclusion criteria for the review.	Yes
Information sources	4	Specify the information sources (e.g., databases, registers) used to identify studies and the date when each was last searched.	Yes
Risk of bias	5	Specify the methods used to assess risk of bias in the included studies.	Yes
Synthesis of results	6	Specify the methods used to present and synthesize results.	Yes
RESULTS			
Included studies	7	Give the total number of included studies and participants and summarise relevant characteristics of studies.	Yes
Synthesis of results	8	Present results for main outcomes, preferably indicating the number of included studies and participants for each. If meta-analysis was done, report the summary estimate and confidence/credible interval. If comparing groups, indicate the direction of the effect (i.e., which group is favoured).	Yes
DISCUSSION			
Limitations of evidence	9	Provide a brief summary of the limitations of the evidence included in the review (e.g. study risk of bias, inconsistency and imprecision).	Yes
Interpretation	10	Provide a general interpretation of the results and important implications.	Yes
OTHER			
Funding	11	Specify the primary source of funding for the review.	Yes
Registration	12	Provide the register name and registration number.	Yes

Page, M.J.; McKenzie, J.E.; Bossuyt, P.M.; Boutron, I.; Hoffmann, T.C.; Mulrow, C.D., et al.The PRISMA 2020 statement: an updated guideline for reporting systematic reviews. *MetaArXiv* **2020**. DOI: 10.31222/osf.io/v7gm2. For more information, visit: www.prisma-statement.org
