# Supplementary material for: Do Lifestyle Interventions in Pregnant Women with Overweight or Obesity Have an Effect on Neonatal Adiposity? A Systematic Review with Meta-Analysis
Source: Nutrients. 2021 Jun 1;13(6):1903. doi: 10.3390/nu13061903 (PMC8228378; doi:10.3390/nu13061903)
Supplement: Supplementary file 1 [file nutrients-13-01903-s001.zip › supplementary/Supplementary_Figure S1.pdf]

Study or Subgroup	Lifestyle intervention Mean	SD	Total	Usual care Mean	SD	Total	Weight	Mean Difference IV, Random, 95% CI
Gallagher et al., 2018	7.89	4.07	105	9.67	4.17	105	42.7%	-1.78 [-2.89, -0.67]
van Horn et al., 2018	10	6	140	12	6	141	26.9%	-2.00 [-3.40, -0.60]
van Poppel et al., 2019	6.4	3.9	80	8.6	4.6	80	30.4%	-2.20 [-3.52, -0.88]
**Total (95% CI)**			**325**			**326**	**100.0%**	**-1.97 [-2.70, -1.24]**

Heterogeneity: $Tau^2 = 0.00$; $Chi^2 = 0.23$, $df = 2$ ($P = 0.89$); $I^2 = 0\%$
Test for overall effect: $Z = 5.29$ ($P < 0.00001$)

Mean Difference
IV, Random, 95% CI

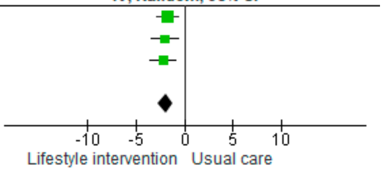

Supplementary Figure S1. Effect of the lifestyle interventions in pregnant women with excessive body weight on gestational weight gain. SD = standard deviation; CI = confidence interval; Tau^2 = Tau-squared test; Chi^2 = Chi-squared test; Df = difference; P = *p* value; I^2 = heterogeneity; Z = Z test [1–3].

1. Gallagher, D.; Rosenn, B.; Toro-Ramos, T.; Paley, C.; Gidwani, S.; Horowitz, M.; Crane, J.; Lin, S.; Thornton, J.C.; Pi-Sunyer, X. Greater Neonatal Fat-Free Mass and Similar Fat Mass Following a Randomized Trial to Control Excess Gestational Weight Gain. Obesity **2018**, 26, 578-587.
2. van Horn, L.; Peaceman, A.; Kwasny, M.; Vincent, E.; Fought, A.; Josefson, J.; Spring, B.; Neff, L.M.; Gernhofer, N. Dietary Approaches to Stop Hypertension Diet and Activity to Limit Gestational Weight: Maternal Offspring Metabolics Family In-tervention Trial, a Technology Enhanced Randomised Trial. Am. J. Prev. Med. **2018**, 55, 603-614.
3. van Poppel, M.N.M.; Simmons, D.; Devlieger, R.; van Assche, F.A.; Jans, G.; Galjaard, S.; Corcoy, R.; Adelantado, J.M.; Dunne, F.; Harreiter; J.; et al. A reduction in sedentary behaviour in obese women during pregnancy reduces neonatal adiposity: the DALI randomised controlled trial. Diabetologia **2019**, 62, 915-925.
