# Supplementary material for: Do Lifestyle Interventions in Pregnant Women with Overweight or Obesity Have an Effect on Neonatal Adiposity? A Systematic Review with Meta-Analysis
Source: Nutrients. 2021 Jun 1;13(6):1903. doi: 10.3390/nu13061903 (PMC8228378; doi:10.3390/nu13061903)
Supplement: Supplementary file 1 [file nutrients-13-01903-s001.zip › supplementary/Supplementary_Figure S2.pdf]

Study or Subgroup	Lifestyle intervention Mean	SD	Total	Usual care Mean	SD	Total	Weight	Mean Difference IV, Random, 95% CI
Gallagher et al., 2018	3,373	587	97	3,235	532	99	31.2%	138.00 [-18.93, 294.93]
van Horn et al., 2018	3,244	489	130	3,213	524	121	37.7%	31.00 [-94.63, 156.63]
van Poppel et al., 2019	3,480	504	80	3,581	510	80	31.1%	-101.00 [-258.12, 56.12]
**Total (95% CI)**			**307**			**300**	**100.0%**	**23.26 [-102.73, 149.24]**

Heterogeneity: Tau² = 6847.61; Chi² = 4.47, df = 2 (P = 0.11); I² = 55%
Test for overall effect: Z = 0.36 (P = 0.72)

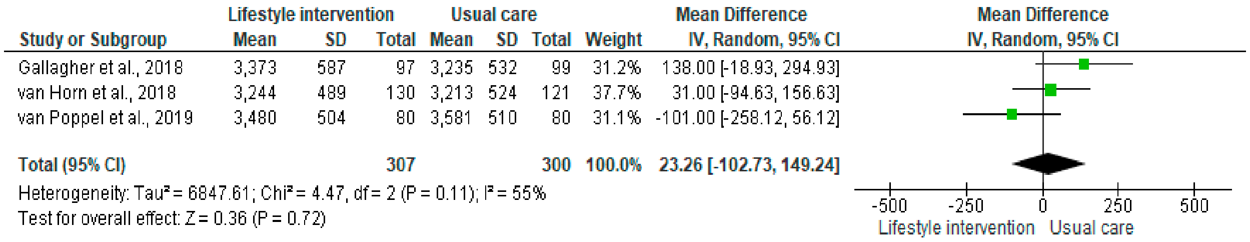

Supplementary Figure S2. Effect of the lifestyle interventions in pregnant women with excessive body weight on birth weight. SD = standard deviation; CI = confidence interval; Tau^2 = Tau-squared test; Chi^2 = Chi-squared test; Df = difference; P = *p* value; I^2 = heterogeneity; Z = Z test. [1–3].

1. Gallagher, D.; Rosenn, B.; Toro-Ramos, T.; Paley, C.; Gidwani, S.; Horowitz, M.; Crane, J.; Lin, S.; Thornton, J.C.; Pi-Sunyer, X. Greater Neonatal Fat-Free Mass and Similar Fat Mass Following a Randomized Trial to Control Excess Gestational Weight Gain. Obesity **2018**, 26, 578-587.
2. van Horn, L.; Peaceman, A.; Kwasny, M.; Vincent, E.; Fought, A.; Josefson, J.; Spring, B.; Neff, L.M.; Gernhofer, N. Dietary Approaches to Stop Hypertension Diet and Activity to Limit Gestational Weight: Maternal Offspring Metabolics Family In-tervention Trial, a Technology Enhanced Randomised Trial. Am. J. Prev. Med. **2018**, 55, 603-614.
3. van Poppel, M.N.M.; Simmons, D.; Devlieger, R.; van Assche, F.A.; Jans, G.; Galjaard, S.; Corcoy, R.; Adelantado, J.M.; Dunne, F.; Harreiter; J.; et al. A reduction in sedentary behaviour in obese women during pregnancy reduces neonatal adiposity: the DALI randomised controlled trial. Diabetologia **2019**, 62, 915-925.
