# Supplementary material for: Do Lifestyle Interventions in Pregnant Women with Overweight or Obesity Have an Effect on Neonatal Adiposity? A Systematic Review with Meta-Analysis
Source: Nutrients. 2021 Jun 1;13(6):1903. doi: 10.3390/nu13061903 (PMC8228378; doi:10.3390/nu13061903)
Supplement: Supplementary file 1 [file nutrients-13-01903-s001.zip › supplementary/Supplementary_Figure S3.pdf]

Study or Subgroup	Lifestyle intervention Mean	SD	Total	Usual care Mean	SD	Total	Weight	Mean Difference IV, Random, 95% CI
Dodd et al., 2016	3,026.64	339.96	488	3,030.07	362.54	482	51.5%	-3.43 [-47.67, 40.81]
Gallagher et al., 2018	2,871	404	95	2,786	405	96	24.4%	85.00 [-29.73, 199.73]
van Poppel et al., 2019	3,029	351	68	3,111	339	67	24.0%	-82.00 [-198.40, 34.40]
**Total (95% CI)**			**651**			**645**	**100.0%**	**-0.68 [-72.87, 71.51]**

Heterogeneity: $Tau^2 = 2122.85$; $Chi^2 = 4.03$, df = 2 (P = 0.13); $I^2 = 50\%$
Test for overall effect: Z = 0.02 (P = 0.99)

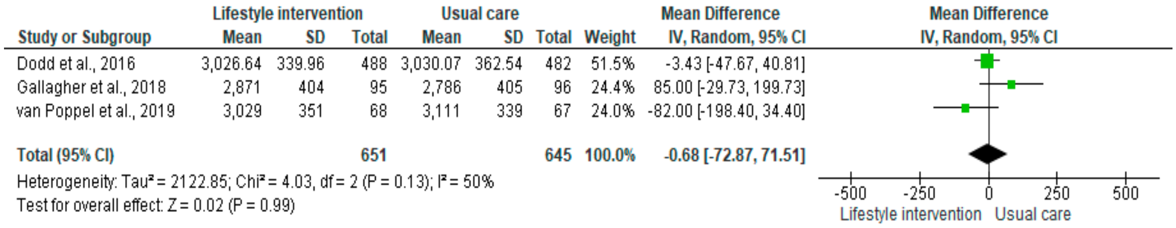

Supplementary Figure S3. Effect of the lifestyle interventions in pregnant women with excessive body weight on the fat-free mass of the newborn. SD = standard deviation; CI = confidence interval; Tau^2 = Tau-squared test; Chi^2 = Chi-squared test; Df = difference; P = *p* value; I^2 = heterogeneity; Z = Z test. [1–3].

1.Dodd, J.M.; Deussen, A.R.; Mohamad, I.; Rifas-Shiman, S.L.; Yelland, L.N.; Louise, J.; McPhee, A.J.; Grivell, R.M.; Owens, J.A.; Gillman, M.W.; Robinson, J.S. The effect of antenatal lifestyle advice for women who are overweight or obese on secondary measures of neonatal body composition: the LIMIT randomised trial. BJOG. **2016**, 123, 244-53.

2. Gallagher, D.; Rosenn, B.; Toro-Ramos, T.; Paley, C.; Gidwani, S.; Horowitz, M.; Crane, J.; Lin, S.; Thornton, J.C.; Pi-Sunyer, X. Greater Neonatal Fat-Free Mass and Similar Fat Mass Following a Randomized Trial to Control Excess Gestational Weight Gain. Obesity **2018**, 26, 578-587.

3. van Poppel, M.N.M.; Simmons, D.; Devlieger, R.; van Assche, F.A.; Jans, G.; Galjaard, S.; Corcoy, R.; Adelantado, J.M.; Dunne, F.; Harreiter; J.; et al. A reduction in sedentary behaviour in obese women during pregnancy reduces neonatal adiposity: the DALI randomised controlled trial. Diabetologia **2019**, 62, 915-925.
