# Supplementary material for: Do Lifestyle Interventions in Pregnant Women with Overweight or Obesity Have an Effect on Neonatal Adiposity? A Systematic Review with Meta-Analysis
Source: Nutrients. 2021 Jun 1;13(6):1903. doi: 10.3390/nu13061903 (PMC8228378; doi:10.3390/nu13061903)
Supplement: Supplementary file 1 [file nutrients-13-01903-s001.zip › supplementary/Supplementary_Figure S4.pdf]

Certainty Assessment							Summary of Findings				
Participants (studies) Follow up	Risk of bias	Inconsistency	Indirectness	Imprecision	Publication bias	Overall certainty of evidence	Study event rates (%)		Relative effect (95% CI)	Anticipated absolute effects	
							With Usual care	With Lifestyle intervention		Risk with Usual care	Risk difference with Lifestyle intervention
**The effect of the lifestyle intervention with overweight/obese pregnant women in relation to neonatal adiposity**											
1494 (4 RCTs)	very serious [a,b]	not serious	not serious	not serious	none	⊕⊕◯◯ LOW	737	757	-	The mean the effect of lifestyle intervention with overweight/obese pregnant women on neonatal adiposity was **0**	MD **1 higher** (0.92 lower to 0.5 higher)
**Birth weight**											
607 (3 RCTs)	serious [a,b]	not serious	not serious	not serious	none	⊕⊕⊕◯ MODERATE	300	307	-	The mean birth weight was **0**	MD **1 higher** (102.73 lower to 149.24 higher)
**Gestational weight gain**											
651 (3 RCTs)	serious [a,b]	not serious	not serious	not serious	none	⊕⊕⊕◯ MODERATE	326	325	-	The mean gestational weight gain was **0**	MD **1 higher** (1.24 lower to 2.7 lower)
**Body fat-free mass**											
1296 (3 RCTs)	serious [a,b]	not serious	not serious	not serious	none	⊕⊕⊕◯ MODERATE	645	651	-	The mean fat-free mass was **0**	MD **1 higher** (72.87 lower to 71.51 higher)

Supplementary Figure S4. Summary of findings: The effect of the lifestyle intervention with overweight/obese pregnant women in relation to the neonatal adiposity.
